# Chemistry and Biological Activities of Cannflavins of the Cannabis Plant

**DOI:** 10.1089/can.2023.0128

**Published:** 2023-12-04

**Authors:** Maged S. Abdel-Kader, Mohamed M. Radwan, Ahmed M. Metwaly, Ibrahim H. Eissa, Arno Hazekamp, Mahmoud A. ElSohly

**Affiliations:** ^1^ Department of Pharmacognosy, College of Pharmacy, Prince Sattam Bin Abdulaziz University, Al-Kharj, Saudi Arabia.; ^2^ Department of Pharmacognosy, Faculty of Pharmacy, Alexandria University, Alexandria, Egypt.; ^3^ National Center for Natural Products Research, School of Pharmacy, University of Mississippi, University, Mississippi, USA.; ^4^ Department of Pharmacognosy and Medicinal Plants, Faculty of Pharmacy (Boys), Al-Azhar University, Cairo, Egypt.; ^5^ Department of Pharmaceutical Medicinal Chemistry & Drug Design, Faculty of Pharmacy (Boys), Al-Azhar University, Cairo, Egypt.; ^6^ Hazekamp Herbal Consulting, Leiden, The Netherlands.; ^7^ Department of Pharmaceutics and Drug Delivery, School of Pharmacy, University of Mississippi, University, Mississippi, USA.

**Keywords:** cannflavins, cannabis, biological activity, chemistry

## Abstract

**Background:** Throughout history, Cannabis has had a significant influence on human life as one of the earliest plants cultivated by humans. The plant was a source of fibers used by the oldest known civilizations. Cannabis was also used medicinally in China, India, and ancient Egypt. Delta-9-tetrahydrocannabinol (Δ^9^-THC), the main psychoactive compound in the plant was identified in 1964 followed by more than 125 cannabinoids. More than 30 flavonoids were isolated from the plant including the characteristic flavonoids called cannflavins, which are prenylated or geranylated flavones.

**Material and Methods:** In this review, the methods of extraction, isolation, identification, biosynthesis, chemical synthesis, analysis and pharmacological activity of these flavonoids are described.

**Results:** The biosynthetic routes of the cannflavins from phenylalanine and malonyl CoA as well as the microbial biotransformation are also discussed. Details of the chemical synthesis are illustrated as an alternative to the isolation from the plant materials along with other possible sources of obtaining cannflavins. Detailed methods discussing the analysis of flavonoids in cannabis are presented, including the techniques used for separation and detection. Finally, the various biological activities of cannflavins are reviewed along with the available molecular docking studies.

**Conclusion:** Despite the low level of cannflavins in cannabis hamper their development as naturally derived products, efforts need to be put in place to develop high yield synthetic or biosynthetic protocols for their production in order for their development as pharmaceutical products.

## Introduction

Cannabaceae is a relatively small family of flowering plants known also as the hemp family. The family was placed under the order Urticales till the 1990s when it was moved to the order Rosales based on molecular data.^1^ The genus *Cannabis* is one of the disputed genera where three species could be recognized: *Cannabis sativa*, *Cannabis indica*, and *Cannabis ruderalis*. Other scientists did not recognize *C. ruderalis* as independent species and included it within *C. sativa*. Alternatively, all three species may be treated as subspecies of *C. sativa*, which is the current thinking.^2^ Cannabis is an annual dioecious herb characterized by leaves with serrate leaflets.^3^ Very early the Chinese distinguished and described male and female cannabis.^4^ Although cannabis is grown in many different localities worldwide, scientific evidence indicated that the origin of the plant is Central Asia.^5,6^

Cannabis is one of the plants with a great impact on human life throughout history. In fact, it is one of the earliest plants cultivated by man. First historical evidence indicated that the plant was cultivated for its fibers in China since 4000 B.C.^7^ The plant seeds were also used by the ancient Chinese for the management of rheumatic pain, intestinal constipation, female reproductive system disorders, and malaria.^8^ Reports indicated that the founder of Chinese surgery “Hua T'o” administered cannabis with wine to anesthetize patients during surgical operations.^7^

An important milestone in cannabis history was the identification of the chemical structure of Δ^9^-tetrahydrocannabinol (Δ^9^-THC), the main psychoactive compound in the plant, by Gaoni and Mechoulam.^9^ Later >125 cannabinoids were identified from the plant.

Besides the biomarker cannabinoids, cannabis also biosynthesizes terpenes responsible for the characteristic plant odor and flavor. More than 100 terpenes have been identified from *C. sativa* growing in different localities and at different stages of the plant's growth.^10–12^ The most common among *C. sativa* terpenes are limonene, *α*-pinene, *β*-myrcene, and *β-*caryophyllene. The presence of terpenes could potentiate the physiological effects of cannabinoids.^13^ Combinations of cannabinoids and terpenes could be an important therapeutic tool and may explain the potential of cannabis to relieve certain symptoms.^14^

Flavonoids are another major class of compounds that were isolated from *C. sativa*. More than 30 flavonoids belonging to 7 basic chemical structures can be glycosylated (C- or O-glycosides), prenylated, geranylated, or methylated. Orientin, vitexin, isovitexin, apigenin, luteolin, kaempferol, and quercetin derivatives constitute the cannabis flavonoids.^14,16^ The presence of flavonoids was monitored in various organs, and the concentration was different from one part to another. For example, the content was 0.07–0.14% (based on dry weight) in cannabis inflorescence and 0.34–0.44% in the leaves.^17^ Cannflavins are prenylated methoxyluteolin derivatives.

**FIG. 1.**
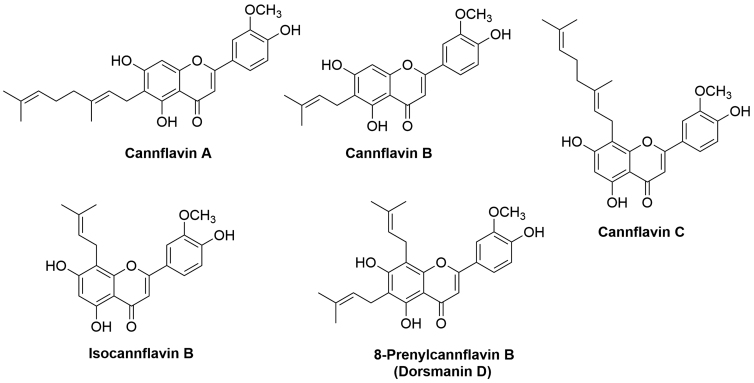
Chemical structures of the known cannflavins.

Four cannflavins have been isolated from cannabis, namely cannflavin A, cannflavin B, cannflavin C, and isocannflavin B (Fig. 1). Analysis of six different varieties of fiber-type hemp female inflorescence samples indicated that cannflavin A was the main compound in almost all the samples. It is reported that the biological activity of cannabinoids can be enhanced in the presence of flavonoids due to a synergistic action or improving their pharmacokinetics.^18^

Interestingly, some flavonoids and biflavonoids expressed potential psychoactive effects *via* significant binding with rat benzodiazepine, dopamine transporter, GABAA, norepinephrine transporter, and sigma-2 receptors.^19^ A pharmaceutical formula containing cupressuflavone isolated from *Juniperus sabina* aerial parts administered via the intranasal route markedly reduced the spontaneous motor activity, motor coordination, and balance of rats using the activity cage and rotarod, respectively.^20^ These findings may indicate some role of flavonoids in cannabis activity. In this review, the isolation, biosynthesis, analysis, and biological activities of cannflavins from cannabis are discussed in detail.

## Isolation of Cannflavins

In 1980, Crombie et al. isolated two O-methoxylated flavonoids for the first time from Thailand-grown cannabis, but the details of the extraction and isolation were not reported. The isolated flavones were identified as geranylated and prenylated chrysoeriols using mass spectrometry, ultraviolet (UV)-shift agents, and ^1^H-NMR (nuclear magnetic resonance).^21^ Two years later, the same research team re-isolated the two compounds and named them canniflavone 1 and canniflavone 2. The dried leaves were subsequently extracted with methylene chloride, *n*-hexane, and ether, and then, the three extracts were combined and evaporated till dryness at 40°C.

The dried combined extract was then dissolved in ether and extracted by a 2% aqueous solution of NaOH followed by acidification with H_2_SO_4_. The crude acid extract was chromatographed on a silica gel column in nylon tubing eluted with a mixture of ether:*n*-hexane (3:1), and the column was divided into 10 bands based on colors. Bands 3 and 4 were found to be rich in flavones, and each was purified by silica gel preparative thin layer chromatography (PTLC) eluted with ether:*n*-hexane (3:1), followed by MeOH:CHCl_3_ (5:95), and finally purified by C_18_-high-performance liquid chromatography (HPLC) using 90% MeOH/H_2_O as eluent to give canniflavone 1 and canniflavone 2. Both compounds were crystallized from MeOH as pale yellow needles, and their chemical structures were elucidated using a combination of spectroscopic techniques (infrared [IR], UV, ^1^H-NMR, and mass spectrometry).^22^

The same two compounds were independently isolated in 1985 from the cannabinoid-free ethanolic extract of *C. sativa* and named cannflavin A (geranylated derivative) and cannflavin B (prenylated derivative) (Fig. 1). The dried leaves were macerated in petroleum ether to remove the cannabinoids and then extracted with ethanol. The ethanolic extract was purified on repeated Si-PTLC. The two cannflavins were chemically identified by ^1^H-NMR and ^13^C-NMR spectroscopic techniques along with UV, IR, and high-resolution electron impact mass spectroscopy (HREIMS) as well as chemical derivatization.^23,24^

In 2004, Choi et al. described the isolation and the extensive NMR elucidation of the chemical structure of cannflavins A and B from the MeOH:CHCl_3_ (1:1) extract of *C. sativa* flowers. The dried extract was redissolved in 90% MeOH and then partitioned with *n*-hexane. The hexane extract was chromatographed using HP-20 resin, followed by silica gel, and finally, Sephadex LH-20 columns to yield cannflavins A and B. Their chemical structures were determined using ^1^H, ^13^C, heteronuclear multiple quantum correlation, heteronuclear multiple bond correlation (HMBC), and nuclear overhauser effect spectroscopy NMR spectroscopic analysis.^25^

Cannflavin C was isolated for the first time in 2008 from the dried flower buds of a high-potency variety of *C. sativa* grown at the University of Mississippi, along with cannflavins A and B. The dried plant material was sequentially extracted with hexanes, CH_2_Cl_2_, EtOAc, EtOH, and EtOH:H_2_O (1:1). The CH_2_Cl_2_, EtOAc, and EtOH extracts were combined, and cannflavins A, B, and C were isolated from the combined extract using different chromatographic techniques including vacuum liquid chromatography (VLC), gravity column, flash column, and HPLC. Silica gel, reversed-phase silica gel (RP_18_, C_18_), and Sephadex LH-20 are examples of the stationary phases used in the purification of the three cannflavins. Their chemical structures were confirmed based on 1D and 2D NMR experiments as well as high-resolution electrospray ionization mass spectroscopy (HRESIMS).^26,27^

Isocannflavin B was isolated from *C. sativa* along with cannflavins A, B, and C using flash chromatography *via* bioassay-guided fractionation. Isocannflavin B was coded FBL-03G, and its chemical structure was determined by NMR and mass spectroscopy (MS) spectrometry as the isomer of cannflavin B in which the prenyl moiety is attached to C-8 instead of C-6.^28^

From cannabinoids-free hemp sprouts (Ermo variety), cannflavins A and B were obtained. The dried powdered plant material was extracted with acetone and then subjected to C_18_-VLC, followed by silica gel column chromatography to afford cannflavin A. A larger amount of cannflavin A was isolated from another column fraction after silica gel flash chromatography and crystallization. Cannflavin B was isolated but in an impure form, and it was synthetically prepared using a modified Robinson flavone synthesis.^29,30^

Cannflavin A was isolated from the leaves of hemp by Guo et al. The leaves were extracted by 95% EtOH under reflux, and the dried extract was redissolved in H_2_O and extracted with EtOAc, which was fractionated on silica gel column chromatography and eluted with EtOAc:petroleum ether (20–100%) to give four fractions (A–D). Cannflavin A was obtained from fraction C after purification over a silica gel column chromatography, followed by flash C_18_ column, and finally by preparative reversed-phase HPLC.^31^ Recently, cannflavin B was isolated from the leaves of the Futura 75 cultivar of industrial hemp.

The dried methanolic extract of the dried leaves was dissolved in 10% aqueous methanol and partitioned against *n*-hexane to give an *n*-hexane extract. The water content of the MeOH extract was increased to 40% and partitioned with CHCl_3_, and the lower organic layer was separated and dried to give CHCl_3_ extract. The CHCl_3_ extract was subjected to droplet counter current chromatography with a mixture of CHCl_3_:MeOH:H_2_O (7:13:8) to give 17 fractions. Cannflavin B was isolated from Fraction 11 after C_18_-HPLC using MeOH:H_2_O:trifluoro acetic acid (TFA) (70:30:0.1) as the mobile phase, and its chemical structure was determined based on 1D and 2D NMR analysis.^32,33^

In 2022, Puopolo et al. reported the gram-scale preparation of cannflavin A from mature hemp. The ground leaves and flowers (300 kg) were extracted with 80% aqueous EtOH, followed by fractionation over HPD700 resin column chromatography eluted with 53% aqueous EtOH. The cannflavin A-rich fraction was purified by dissolving in hexanes and filtration, followed by crystallization from EtOAc:acetone mixture (5:1) at 4°C to yield 38.7 g of cannflavin A.^34^

Cannflavin A, cannflavin B, and 8-prenyl derivative of cannflavin B were also isolated from plants other than cannabis. Cannflavin A was obtained from *Mimulus bigelovii*. The EtOH extract was successively fractionated with CH_2_Cl_2_, EtOAc, and *n*-butanol. Cannflavin A was isolated from the CH_2_Cl_2_ fraction by applying several column chromatographic techniques including flash column over silica gel, Sephadex LH-20 column, and C_18_ HPLC. IR, HRESIMS, ^1^H-NMR, and^13^C-NMR spectroscopy were used to determine the chemical structure of cannflavin A.^35^

Cannflavin B and 8-prenyl cannflavin B (dorsmanin D) were isolated from twigs of *Dorstenia mannii*. The plant was extracted with MeOH:CH_2_Cl_2_ (1:1) and MeOH. The two extracts were combined and dried and then were subjected to partition extraction CHCl_3_ and EtOAc. The two fractions were also combined based on TLC and chromatographed on a silica gel column gradually eluted with *n*-hexane, EtOAc, and EtOAc/MeOH mixtures to afford 50 fractions. The combined polar column fractions (Fractions 37–50) were further purified using Sephadex LH-20 and successive PTLC to afford cannflavin B and its 8-prenyl derivative.^36^

## Biosynthetic Pathway and Biotransformation

### Biosynthetic pathway of cannflavins

**FIG. 2.**
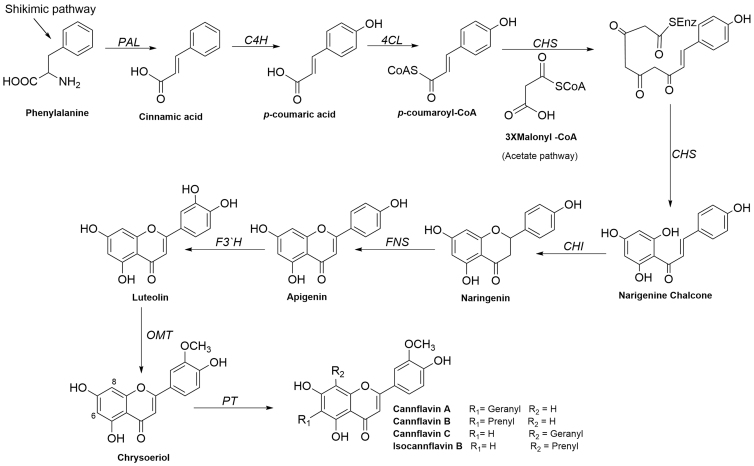
Biosynthetic pathways for cannflavins A, B, and C and isocannflavin B.

Phenylalanine and malonyl CoA are the building blocks or the precursors in the biosynthesis of cannflavins. Phenylalanine is derived from the shikimate pathway, whereas malonyl CoA is biosynthesized from the acetate pathway.^37^ The first step in the biosynthesis of cannflavins is the formation of cinnamic acid from phenylalanine by the enzyme phenylalanine ammonia-lyase (PAL) (Fig. 2), followed by the hydroxylation of cinnamic acid to *p*-coumaric acid by cinnamate-4-hydroxylase (C4H) enzyme, which is activated to *p*-coumaroyl-CoA by 4-coumarate: CoA ligase (4CL). Chalcone synthase (CHS) enzyme catalyzes the condensation of one molecule of *p*-coumaroyl-CoA with three molecules of malonyl CoA to yield naringenin chalcone (Fig. 2).

Ring closure of the intermediate naringenin chalcone by the enzyme chalcone isomerase (CHI) results in the formation of naringenin (the starting compound for the biosynthesis of flavones and flavonols). Flavone synthase (FNS) introduces a double bond between C2 and C3 of naringenin to form apigenin, which is in turn hydroxylated at C-4′ by flavonoid 3′-hydroxylase (F3′H) to yield luteolin. Methylation of luteolin by *O*-methyltransferase (OMT) enzyme to chrysoeriol followed by prenylation or geranylation of ring A affixed at C-6 or C-8 finally results in the formation of cannflavins.^13,38–40^

Prenylation and geranylation occur by prenyltransferase enzyme (PT), which introduces a geranyl moiety (C_10_H_16_) to C-6 to produce cannflavin A or a prenyl moiety to the same carbon (C-6) to give cannflavin B. Geranylation at C-8 forms cannflavin C, whereas prenylation at C-8 yields isocannflavin B (Fig. 1). In 2019, Rea et al. identified the methyltransferase and PTs involved in the biosynthesis of cannflavins as CsOMT21 and CsPT3, respectively, by using both phylogenomic and biochemical approaches.^38^

### Biotransformation of cannflavins

**FIG. 3.**
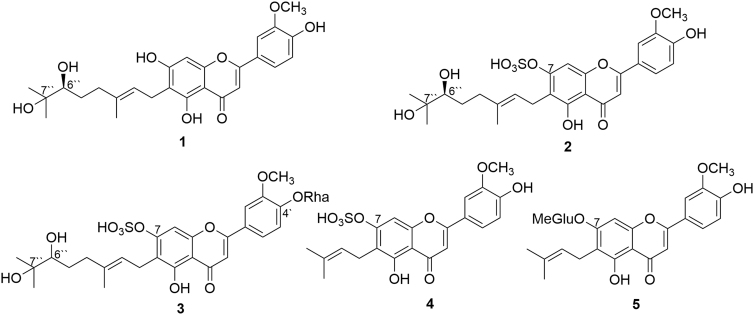
Chemical structure of cannflavin A and cannflavin B metabolites.

Five metabolites (**1–5**) were isolated and identified as a result of the microbial metabolism of cannflavins A and B by the two fungi, *Mucor ramannianus* (ATCC 9628) and *Beauveria bassiana* (ATCC 13144). *M. ramannianus* converted cannflavin A to three metabolites, which were identified as 6″S,7″-dihydroxycannflavin A (**1**), 6″S,7″-dihydroxycannflavin A 7-sulfate (**2**), and 6″S,7″-dihydroxycannflavin A 4′-*O*-α-l-rhamnopyranoside (**3**). *B. bassiana* transformed cannflavin B to cannflavin B 7-sulfate (**4**) and cannflavin B 7-*O*-β-D-4″′-*O*-methylglucopyranoside (**5**). The chemical structures (Fig. 3) of the five isolated metabolites were determined using HRESIMS and NMR spectroscopic methods.^41^

## Chemical Synthesis

The identification of cannflavins has generated significant interest in their synthesis, as they have demonstrated potential therapeutic applications and their low abundance has hampered their isolation in sufficient quantities for further research. For example, Puopolo et al. used 300 kg of dried hemp powder to obtain only 38.73 g of cannflavin A.^34^

Chemical synthesis presents several advantages for the production of cannflavins. A fundamental advantage is the ability to produce these compounds in large quantities, which is critical for extensive study of their therapeutic potential. Moreover, chemical synthesis offers the opportunity to obtain highly pure cannflavins, which is advantageous for research and clinical applications. Furthermore, chemical synthesis provides a means to modify the chemical structure of cannflavins, which is essential for optimizing their therapeutic properties. This approach can result in the creation of novel compounds with improved activity and/or selectivity.

In particular, chemical modification can enhance the solubility, bioavailability, and stability of cannflavins. In addition, chemical synthesis offers the potential to produce cannflavins that are not naturally occurring, which can provide valuable insights into the structure–activity relationships of these compounds. Such insights can facilitate the development of novel therapeutics that possesses enhanced efficacy, safety, and tolerability. Minassi et al. reported the use of a regioselective method of synthesis to obtain cannflavin B and isocannflavin B.^30^ In another study, genetically engineered strains of the yeast *Saccharomyces cerevisiae* were utilized to obtain cannflavin A, cannflavin B, isocannflavin A, and isocannflavin B.^42^

**FIG. 4.**
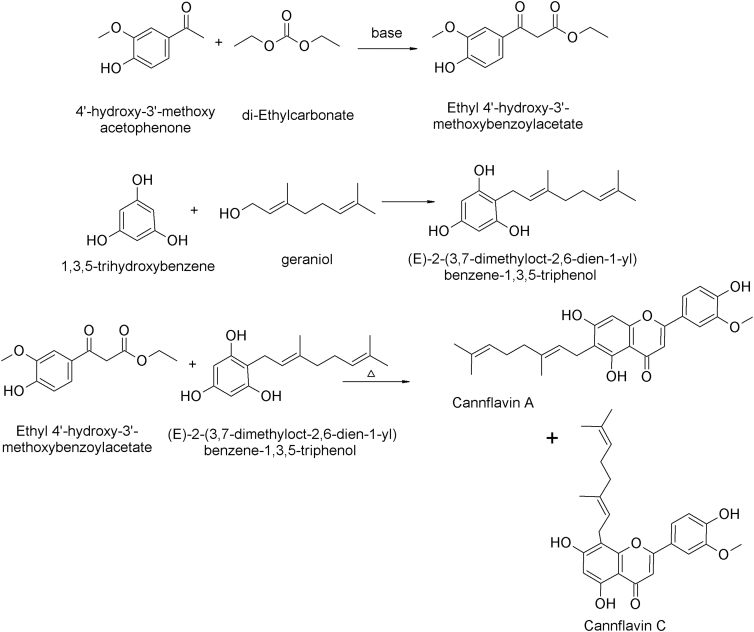
Chemical synthesis of cannflavin A and cannflavin C.

A low-cost method for the synthesis of cannflavin A and/or cannflavin C that is characterized by its ease and availability of the raw materials is reported.^43^ The method involves few reaction steps and has a short production time, making it a highly efficient process. As illustrated in Figure 4, the first step involves the condensation of 4′-hydroxy-3′-methoxyacetophenone and diethyl carbonate under basic conditions, resulting in the formation of ethyl 4′-hydroxy-3′-methoxybenzoyl acetate. In the second step, 1,3,5-trihydroxybenzene is reacted with geraniol to produce (*E*)-2-(3,7-dimethyloct-2,6-dien-1-yl)benzene-1,3,5-triol, which serves as the precursor for the final step. The last step involves the high-temperature condensation of ethyl-4′-hydroxy-3′-methoxybenzoyl acetate and (*E*)-2-(3,7-dimethyloct-2,6-dien-1-yl)benzene-1,3,5-triol to yield cannflavin A and cannflavin C. The resulting mixture can then be subjected to a separation and purification process to obtain highly pure compounds.

## Analysis of Cannflavins

### Localization

Flavonoids are produced primarily in cannabis leaves, and to a lesser extent in inflorescences,^17^ but they are also detectable in roots and seeds. Sprouting of cannabis seeds can induce the production of cannflavin A and cannflavin B, while not triggering the production of cannabinoids.^29^ Cannabis leaves may contain about 1% total flavonoids, especially apigenin and quercetin. Only a small proportion of this consists of cannflavins.^44^

The distributions of flavonoids and cannabinoids in cannabis tissues are different, and there is no indication that the trichomes are involved in the biosynthesis of flavonoids.^45^ In addition to tissue-specific distribution, flavonoid profiles were also shown to vary over time during plant maturation. As many flavonoids possess protective functions for plants, their production is typically responsive to environmental factors, which is also observed in cannabis.^40^

### Extraction and yield

While simple chemical methods may be used for the estimation of total flavonoids content, more sophisticated chromatographic separation and spectrometric analysis must be employed for the identification and quantification of individual compounds present. The extraction of flavonoids from the source materials is typically the first step involved in their analysis. Because flavonoids are hydrophilic, they are usually extracted with polar solvents such as methanol, ethanol, or acetone.

A recent review article on cannflavins^46^ identified 12 studies that provide detailed information on cannflavin extraction and yield. Extraction methods reported in the review included:

- Repeated maceration with petroleum spirit and subsequent maceration with 100% ethanol.
- Initial extraction with chloroform:methanol solution; partitioning with *n*-hexane; then dissolve in ethanol.
- Initial percolation with EtOH, followed by fractionation of the resulting extract between dichloromethane and water.
- Sequential extraction with hexanes, dichloromethane, ethyl acetate, ethanol, and water.
- Extraction with acetone two times.
- Dynamic maceration with *n*-hexane, dichloromethane, ethyl ether, and toluene.
- Extraction with methanol in an ultrasonic bath.

The review article also summarized the different yields reported for the three cannflavins (from dry plant material by weight):

- Cannflavin A—range: 0.000013–0.019%; *n*=7 studies
- Cannflavin B—range: 0.00055–0.0064%; *n*=4 studies
- Cannflavin C—0.00014%; *n*=1 study

These data indicate that cannflavins are typically present in cannabis materials in very low concentrations only.

### Pre-extraction of cannabinoids

The total concentration of flavonoids in cannabis is very low compared with the content of cannabinoids. To reduce possible chromatographic interference from cannabinoids in the concentrated extract, a sample pretreatment may be considered. This is based on the principle that, in contrast to many other phenolics, cannabinoids easily dissolve in nonpolar solvents, such as hexane, toluene, or petroleum ether.

This principle was applied in one of the earliest studies on cannflavins analysis. Barret et al. isolated cannflavin A and cannflavin B from cannabis as part of a study looking for inhibitors of prostaglandin E2 production by cultured rheumatoid synovial cells. The air-dried powdered leaves were repeatedly macerated with petroleum spirit (40–60°C) until cannabinoids were removed (as indicated by Fast Blue Salt B staining). The residual material was air-dried and then repeatedly macerated in 100% ethanol. The resulting extract was concentrated under a vacuum and shown to be free from cannabinoids. Using a series of preparative TLC methods, cannflavin A and cannflavin B could be isolated.^23,24^

A study by Peschel and Politi describes pretreating cannabis by extraction with heptane to remove cannabinoids. The remaining plant material was then extracted with more polar solvents including methanol. Using this method, cannflavin content was 2.6% of the total cannabinoid content (CAN_tot_) in crude (non-pretreated) extracts but increased to 13.9% of CAN_tot_ in cannabinoid-reduced extracts. The study concluded that further enrichment of cannflavins without parallel concentrations of cannabinoids (or other phenolics) may only be achieved with more sophisticated fractionation and chromatographic techniques.^47^

In another study by Pellati et al., the solvents ethanol, acetone, and ethyl acetate were compared for extracting cannflavins from cannabis flowers.^18^ Acetone was found to give the best results. Next, cannabis flowers were pre-extracted three times with *n*-hexane. After this treatment, the residual plant material was extracted three times with acetone by means of dynamic maceration. It was found that *n*-hexane pretreatment was able to remove most cannabinoids, without reducing the amount of cannflavins extracted. In contrast, various other nonpolar solvents tested in the same study led to a decrease in the final amount of flavonoids extracted from cannabis. The pretreatment did not cause degradation of the compounds of interest nor the formation of artifacts.

In the same study, the authors tried the decarboxylation process of the plant material (before the pre-maceration with *n*-hexane) to convert the more polar cannabinoid acids into their neutral less polar counterparts, which are more soluble in this nonpolar solvent. However, the peak areas related to flavonoids decreased after the heating process, and the removal of cannabinoids was not significantly increased; therefore, decarboxylation was not recommended.

### Separation and detection

For the identification and quantification of individual cannflavins, chromatographic separation in combination with spectroscopic detection is needed. Separation is typically achieved by using HPLC methodology coupled with UV or MS detection. Chromatographic data for all cannflavins have been reported in detail, including UV spectra, MS fragmentation, as well as ^1^H and ^16^C NMR data.

For laboratory analysis, analytical standards of cannflavins A, B, and C are available. In one study, the amounts of cannflavins A and B in cannabis samples were determined against a calibration curve of chrysoeriol, having the same chromophore, and the content was corrected by using the molecular weight ratio.^18^

HPLC separation has been performed using various systems, including a reversed-phase (C_21_) column in combination with a solvent gradient consisting of acetonitrile/water/formic acid,^18,33,38^ a phenyl column with a gradient of acetonitrile/H_2_O/TFA,^47,48^ or a biphenyl column with a methanol/water/formic acid gradient.^49^

The UV spectra for all the three cannflavins are similar and show an absorbance maximum of around 275 and 345 nm, and as a result, various wavelengths may be selected for the UV detection of cannflavins (Table 1).^23,24,26,34^

**Table 1.** Ultraviolet Characterization of Cannflavins A, B, and C

	***λ*_max_ (log *ɛ*)**	***λ*_max_ (log *ɛ*)**
Cannflavin A	274 nm (4.20)	344 nm (4.33)
Cannflavin B	278 nm (4.08)	346 nm (4.26)
Cannflavin C	275 nm^a^	

^a^ log *ɛ* for cannflavin C was not reported.

Cannabinoids and cannflavins can be detected in a single HPLC run. This is useful for making fingerprints, but the cannabinoid peaks may be overwhelmingly dominant. Two studies applied an HPLC system using a phenyl column, a H_2_O/acetonitrile/TFA gradient, and UV detection at 214 nm for such analysis.^47,48^ Cannflavin B (ca. 12 min) and cannflavin A (ca. 22 min) were eluted before the first cannabinoid peak cannabigerolic acid (CBGA) (ca. 24 min) in a 50 min total run time.

Peschel and Politi also tested the recovery of cannflavin A and cannflavin B during the extraction of cannabis materials with different cannabinoid contents. Recovery rates of only 60.5–88.2% suggest that extraction of cannflavins can be partially hampered when dealing with CBD-rich extracts.^47^

An application note published by Shimadzu in early 2023 describes an HPLC system that can separate 32 cannabinoids and cannflavins A and B in a runtime of 32.5 min. The system uses a C_18_ column, in combination with a solvent gradient consisting of solvent A: H_2_O, 8% (v/v) MeOH, 0.035% (v/v) formic acid, 1.8 mM ammonium formate, and solvent B: acetonitrile. UV detection of cannflavins was performed at 340 nm. Using this method, cannflavin B elutes before all cannabinoids, whereas cannflavin A elutes between cannabidiolic acid and CBGA.^50^

### MS detection

MS detection has always been an important tool in the identification and quantification of natural products, including flavonoids. HREIMS was essential for the initial structure elucidation of cannflavins A and B.^24^ Rea et al. used HPLC coupled to a quadrupole time-of-flight mass spectrometer to elucidate the bioenzymatic pathway of cannflavin production in cannabis plants.^38^ A good overview of the published mass spectrometry data for cannflavins A, B, and C was provided in 2020 by Erridge et al.^46^

Identification of individual phenolic acids and flavonoids was conducted through UHPLC-Q-Orbitrap HRMS. By a combination of MS and MS/MS spectra, a total of 22 different polyphenolic compounds were identified from different samples of cannabis inflorescences.^51^ Cannflavins A and B were the most commonly detected flavones, with a mean value of 61.8 and 84.5 mg/kg, respectively. The study showed that levels of cannflavins in different individual plants of the same cannabis variety can fluctuate over a range of 10-fold: for example, cannflavin B ranged from 26.2 to 215.5 mg/kg in variety Tiborszallasi (*n*=7) and from 11.9 to 154.4 mg/kg in variety Carmagnola CS (*n*=4).

With the recent extremely sensitive MS imaging techniques such as desorption electrospray ionization and matrix-assisted laser desorption ionization, it is possible to analyze the flavonoid content of individual trichomes.^45^

### NMR spectroscopy

NMR analysis has played a crucial role in the structure elucidation of the different cannflavins. Barrett et al. used the ^1^H-NMR spectrum (in combination with the mass spectrum) to first indicate that cannflavin B differed from cannflavin A only in the absence of the five-carbon alkyl unit in the side chain.^24^ Also, cannflavin C was identified based on a small difference in NMR signals compared with cannflavin A. The spectroscopic data were similar to those reported for cannflavin A except for the location of the geranyl group at C-8 instead of C-6.^26^

The NMR signals for cannflavin A and cannflavin B have been reported in several (deuterated) solvents. The first NMR data (in acetone and chloroform) were published by Barrett et al. as part of the initial structure elucidation of both compounds. In 2004, Choi et al. provided detailed ^1^H- (400 MHz) and ^13^C-NMR (100 MHz) assignments for cannflavin A and cannflavin B measured in acetone.^25^ More recently, Rea et al. analyzed the same two compounds at higher field strength for ^1^H (600 MHz) and ^13^C (150 MHz) and reported detailed HMBC correlations. A good summary of the available NMR data for all three cannflavins is provided by Erridge et al.^46^

According to Peschel and Politi, the NMR may also be used to detect cannflavins in mixtures or extracts. For example, cannflavin A exhibits some signals typical for the flavonoid structure and the prenyl moiety, which are distinctive from the signals of the main phytocannabinoids. The aromatic protons at *δ*_H_ 7.55, 6.94, 6.89, and 6.55 ppm may not be selective in mixtures with other flavonoids but quite specific for more lipophilic cannabis extracts where non-prenylated flavonoids are unlikely to be found. The methoxy peak at *δ*_H_ 3.89 ppm may be shared by other flavonoids but is distinguishable from main cannabinoids.^47^

Despite the possibility for identification, the detection of cannflavins in cannabis extracts by ^1^H NMR appears limited due to low concentration and signal intensity in relation to predominant cannabinoids.

## Biological Activities of Cannflavins

### Anti-inflammatory effects

Chronic inflammation can lead to a wide range of serious health problems, including autoimmune diseases, cardiovascular disease, and cancer.^52^ Thus, finding effective anti-inflammatory agents is crucial for maintaining good health. Examples of such potential agents are cannflavins, as they expressed promising anti-inflammatory activities in several studies.

Without showing apparent toxicity, cannflavins were able to inhibit the release of prostaglandin E by a ratio of >90%. Cannflavins' potentialities to inhibit prostaglandin E release from synovial cells were intermediate between aspirin on one side and both indomethacin and dexamethasone on the other side.^23,24^ On the molecular level, cannflavins A and B act as dual inhibitors of two essential inflammatory enzymes, microsomal prostaglandin E synthase-1 and 5-lipoxygenase, in a concentration-independent and reversible manner. Both enzymes are important in the biosynthesis of the proinflammatory mediators, prostaglandin 2 and leukotrienes, respectively. In addition, cannflavin A alone weakly inhibited cyclooxygenases COX-1 and COX-2.^29^ Another study reported a promising anti-lipoxygenase potential of cannflavins without significant inhibition of cyclooxygenase enzymes.^53^

### Neuroprotective effects

Cannflavins demonstrated strong neuroprotective effects, which may be linked to their anti-inflammatory potentialities, in several studies. Lowe et al. reported the potential of a cannabis-based flavonoid pharmaceutical composition containing cannflavins A, B, and C in addition to several other flavonoids to prevent and treat several central nervous system-linked diseases and disorders.^54^

In another study, cannflavin A enhanced the toxicity resistance and viability of neuronal PC12 cells against amyloid *β*-induced cytotoxicity using the MTT assay by reducing A*β*_1_–42 aggregation and fibril formation. In more detail, cannflavin A increased viability by 40% from 1 to 10 μM. The neuroprotective effect of cannflavin A was associated with an inhibition of Aβ1–42 fibrils, neurotoxicity, and aggregate density, as evidenced by electron microscopy.^55^

Cannflavin A exhibited strong therapeutic potential against neurodegenerative and neuroinflammatory diseases through kynurenine-3-monooxygenase inhibition with an half maximal inhibitory concentration (IC_50_) of 29.4 μM, compared with Ro 61-8048, the positive control, (IC_50_=5.1 μM).^33^

### Anticancer activities

Several reports confirmed that cancer is also linked strongly to chronic inflammation.^56^ The anticancer activities of cannflavins were reported in several records. As a part of the cannabis-based pharmaceutical composition, cannflavins A, B, and C exhibited prevention and treatment potential against several types of cancers targeting oncogenic factors, such as kinases, sirtuins, bromodomains, matrix metalloproteinases, and BCL-2.^57^

In addition, cannflavin A exhibited a striking potential against HepG2 and HT-29 cell lines.^31^ In another study, the combined potential of cannflavin A, and gemcitabine or cisplatin resulted in various responses depending on the concentrations and drugs used. Also, cannflavin A induced apoptosis via caspase 3 cleavage and was able to reduce invasion by a ratio of 50%.^58^

In a dose-dependent manner, cannflavins A and B decreased the cell viability of Taxol-resistant breast cancer cell lines without toxicity against the nontumorigenic breast cell line. Cannflavins A and B promoted apoptosis and autophagy and reduced the viability of chemotherapeutic-resistant breast cancer cells. When combined with paclitaxel or cannabidiol, Δ^9^-THC, cannabichromene, cannabivarin, cannflavins A and B produced various responses from antagonistic to additive, and even synergistic, depending on the concentrations used.^59^

Isocannflavin B activated apoptosis in two pancreatic cancer cell lines (Panc-02 and Ptf1/p48-Cre) resulting in delaying tumor progression (local and metastatic) and increasing the affected mice's survival time.^28^ Furthermore, Brunelli et al. reported the potential of isocannflavin B in autophagy, and the removal and regulation of dysfunctional intracellular compositions, in estrogen-sensitive and -insensitive breast cancer cell lines. Isocannflavin B arrested cancer cells growth at a concentration of ≥1 μM during the Gap 0 and Gap 1 phases, without inducing apoptosis. Isocannflavin B also arrested cyclin-dependent kinase inhibitor 1 expression and induced autophagic cell toxicity in the treated breast cancer cell lines.^60^

### Antiviral activity

Cannflavin A exhibited promising *in silico* inhibitory effects against the human immunodeficiency viruses HIV-1 protease^61^ and M^pro^ of 2019-nCoV,^62^ with binding affinity values of −9.7 kcal/mol.

Cannflavin A exhibited remarkable binding affinity values against the protein NS5 protein (NS5 MTase and NS5 RdRp) of Zika virus in addition to four serotypes of the dengue virus.^63^ Cannflavin A is also among the phytochemicals that are predicted to show efficient docking to the helicase (RNA site), helicase (ATP site), methyltransferase, and RNA-dependent RNA polymerase of Zika virus with binding energies of −131.7, −134.6, −126.9, and −120.3 kJ/mol, respectively.^64^

### Antiparasitic activity

In one study, cannflavin A displayed strong *in vitro* antileishmanial activity with an IC_50_ value of 4.5 μg/mL, while it displayed strong antileishmanial activity with an IC_50_ value of 4.5 μg/mL.^26^ In another study, cannflavin A showed moderate antileishmanial and antitrypanosomal activities with IC_50_ values of 14.6±3.7 and 1.9±0.8 μg/mL, respectively.^34^ Cannflavin A also has promising docking energy against *Leishmania major* pteridine reductase (*E*_dock_=−144.0 kJ/mol), comparable to methotrexate, the co-crystallized ligand (*E*_dock_=−149.8 kJ/mol).^65^

### Other activities

Some other biological activities were reported for cannflavins. For example, cannflavins A and C showed moderate antioxidant activities in the 2,2-diphenyl-1-picrylhydrazyl assay.^26^

Also, as a part of a cannabis-based pharmaceutical composition, cannflavins A, B, and C were able to prevent and treat certain ocular diseases and disorders, particularly glaucoma and myopia.^66^

The reported activities of cannflavins are supported by detailed mechanistic studies, *in vivo* experiments, and/or *in silico* evaluation. Such acquired data rule out the possibility of being Pan-assay interference compounds.^67^

## Conclusions

Cannflavins are prenylated or geranylated flavones, mostly known to be part of the chemical components of cannabis. These compounds possess desirable pharmacological activities that are worthy of developing further. However, the low level of cannflavins in cannabis hampers their development as naturally derived products. Efforts need to be put in place to develop high-yield synthetic or biosynthetic protocols for their production in order for their development as pharmaceutical products to materialize.

## Abbreviations Used

1D and 2D NMR: one-, two-dimensional nuclear magnetic resonance
BCL-2: B cell lymphoma 2
C_18_: reversed-phase silica gel-18
C4H: cinnamate-4-hydroxylase
CBD: cannabidiol
CBGA: cannabigerolic acid
CHI: chalcone isomerase
CHS: chalcone synthase
4CL: 4-coumarate: CoA ligase
EI: electron ionization
F3′H: flavonoid 3′-hydroxylase
FNS: flavone synthase
GABA: gamma-aminobutyric acid
HMBC: heteronuclear multiple bond correlation
HPLC: high-performance liquid chromatography
HREIMS: high-resolution electron impact mass spectroscopy
HRESIMS: high-resolution electrospray ionization mass spectroscopy
IR: infrared
MS: mass spectroscopy
OMT: *O*-methyltransferase
PAL: phenylalanine ammonia-lyase
PT: prenyltransferase enzyme
ppm: parts per million
PTLC: preparative thin layer chromatography
Δ^9^-THC: delta-9-tetrahydrocannabinol
TFA: trifluoroacetic acid
UHPLC: ultra high-performance liquid chromatography
UV: ultraviolet
VLC: vacuum liquid chromatography
